# Editorial—Discovery and Valorization of New Food Matrices

**DOI:** 10.3390/foods15030512

**Published:** 2026-02-02

**Authors:** Mattia Spano, Luisa Mannina

**Affiliations:** 1 Department for the Promotion of Human Science and Quality of Life, San Raffaele University, Via di Val Cannuta 247, 00166 Rome, Italy; 2 Department of Chemistry and Technology of Drugs, Sapienza University of Rome, Piazzale Aldo Moro 5, 00185 Rome, Italy; luisa.mannina@uniroma1.it

The relationship between humans and food has always been characterized by continuous and dynamic changes in terms of food choices, production technologies, cooking approaches, and preservation. These changes are related to a wide range of aspects of human history and habits, namely climate [1], technological innovation [2], migration and cultural exchanges [3], economic landscape [4], and health-related food perception [5].

Nowadays, a strong transition towards new food matrices and ingredients is occurring, mainly driven by environmental sustainability and the necessity to cover the whole world population’s requests [6,7]. It is well known that the ever-growing food demand, due to increasing population, and the high environmental impact of intensive food production, mainly in terms of protein sources, are not sustainable anymore.

In this regard, several strategies are used to solve the previously described issues, namely the following: (I) use of new and innovative food matrices; (II) valorization of food waste for new applications; (III) improvement of technological processes to improve nutritional and biological properties of food matrices.

The valorization of new food matrices is currently a primary focus in the Food Science field, with a particular focus on protein sources, although other nutrients (vitamins, minerals, polyunsaturated fatty acids, etc.) or bioactive compounds are also of interest. The search for alternative protein sources is related to the need for more ethical, environmentally sustainable, and low-resource-consuming matrices, with respect to the traditionally used animal protein sources.

In the present Special Issue, some of the published papers have focused on this.

In particular, Ferri et al. (Contribution 1) have used NMR spectroscopy to characterize the metabolomic profile of *Tenebrio molitor*, an edible insect species often proposed as a valid protein alternative. *Tenebrio molitor* samples have been reared on chestnut shell-enriched substrate, using different by-product amounts, and a further characterization of the metabolite profile has been proposed. Moreover, together with the characterization of this insect species, food by-product valorization has also been considered by using chestnut shells as a potential feed source. The obtained results have underlined significant metabolite changes related to chestnut shell, i.e., increments of glucose, acetate, lactate, adenosine, uracil, uridine, glycerol, uridine, ethanolamine, and choline have been observed, together with a decrement of other metabolites, namely trehalose, phosphorylcholine, AMP, and fumarate. This study has underlined that insect feeding can strongly affect their chemical profile, thus suggesting the relevance of an appropriate feeding choice to obtain products with specific nutritional and organoleptic properties.

Together with insects, cyanobacteria represent another example of an innovative protein source with increasing production and application growth occurring in recent years. Among cyanobacteria, *Arthrospira platensis*, mainly known as Spirulina, is the most representative example of the group used as a protein source but also as a source of several bioactive and well-being-related compounds. Although the chemical profile of this matrix has been largely studied, it is well known that any process used to extract bioactive compounds can affect the final extract composition, thus affecting product properties. In the study by Milia et al. (Contribution 2), this has been greatly discussed by proposing ultrasound-assisted extraction (UAE) coupled with low-temperature PBS maceration as a methodology to enhance the nutritional and bioactive properties of Spirulina extracts. Increased yields of lipids, fatty acids, allophycocyanin, phycoerythrin, carotenoids, and polyphenols have been observed, underlining the enhanced antioxidant activity obtained with the developed extraction method.

Although not proposed as a protein source, pot-pollen has been deeply discussed in a review by Vit et al. (Contribution 3). Pot-pollen is the final product obtained by fermentation of the pollen transported in cerumen pots by stingless bees. This process, different from the better-known one carried out by *Apis mellifera*, is responsible for the formation of a product rich in volatile compounds, vitamins, minerals, amino acids, and probiotics. Moreover, it presents a better digestibility with respect to common pollen. These features make pot-pollen a matrix with potential applications in both food and nutraceutical fields and should stimulate an increase in its analysis and composition elucidation.

In the context of food waste valorization, chemical profile characterization and proposal of new applications is now a pivotal approach that contributes to enhancing human nutrition and health and ensuring sustainability and equitable access to nutrients.

Petraru et al. (Contribution 4) have contributed to this field by analyzing the chemical composition of flaxseed oilcake, the residual product of flaxseed oil cold pressing. The study underlined a rich chemical and bioactive profile, making this matrix potentially applicable for several nutritional and nutraceutical applications. In particular, the used multimethodological approach has underlined interesting features of flaxseed oilcake, namely a higher amount of minerals and proteins with respect to flaxseed, as well as the highest antioxidant capacity and phenolic content.

Regarding the application of technological processes to change and improve the nutritional properties of food matrices, Yin et al. (Contribution 5) and Regatieri et al. (Contribution 6) have focused their attention on fruit drying and fruit juices fermentation, respectively.

In particular, Yin et al. dried mulberry fruits with five different drying processes, monitoring the changes in volatile profiles. The obtained results have underlined how the quantitative volatile profile of mulberries is affected by drying, with ester content being higher in the hot-air dried samples, aldehyde content being higher in the vacuum and microwave drying samples, and alcohol content being higher in the fresh samples. Based on these results, it is clear that flavor profile, related to volatile compounds as well as the potential biological activity, can be strongly affected by the drying process, since these molecules are well-known for their health properties.

Another interesting aspect related to fruit processing has been discussed by Regatieri et al., whose study has focused on the development of an NIR approach to monitor the changes in fingerprint profiles of fermented fruit juices, with several bacterial species added as probiotics. Changes during different fermentation times have been observed, with the possibility of creating a predictive model to monitor and standardize the fermentation process to obtain a product with specific organoleptic and nutritional properties.

The Special Issue “Discovery and Valorization of New Food Matrices” has contributed to addressing the research in the field of discovering, valorizing, and characterizing innovative food matrices, food ingredients, and food processes. Research in this field is ever-growing and promising, and its continuation is strongly recommended to achieve a suitable combination of nutritional accessibility, human health, and environmental sustainability.
